# The neural basis of authenticity recognition in laughter and crying

**DOI:** 10.1038/s41598-021-03131-z

**Published:** 2021-12-09

**Authors:** Maciej Kosilo, Mónica Costa, Helen E. Nuttall, Hugo Ferreira, Sophie Scott, Sofia Menéres, José Pestana, Rita Jerónimo, Diana Prata

**Affiliations:** 1 Instituto de Biofísica e Engenharia Biomédica, Faculdade de Ciências da Universidade de Lisboa, Campo Grande 016, 1749-016 Lisbon, Portugal; 2 William James Center for Research, Lisbon, Portugal; 3 Department of Psychology, Lancaster University, Lancaster, UK; 4 Institute of Cognitive Neuroscience, University College London, London, UK; 5 APPsyCI - Applied Psychology Research Center Capabilities & Inclusion, ISPA - Instituto Universitário, Lisbon, Portugal; 6 Departamento de Biociências, ISPA – Instituto Universitário, Lisbon, Portugal; 7 Instituto Universitário de Lisboa (Iscte-IUL), CIS-Iscte, Lisbon, Portugal; 8 Department of Neuroimaging, Institute of Psychiatry, Psychology and Neuroscience, London, UK

**Keywords:** Cognitive neuroscience, Psychology, Human behaviour, Neuroscience, Social neuroscience, Empathy

## Abstract

Deciding whether others’ emotions are genuine is essential for successful communication and social relationships. While previous fMRI studies suggested that differentiation between authentic and acted emotional expressions involves higher-order brain areas, the time course of authenticity discrimination is still unknown. To address this gap, we tested the impact of authenticity discrimination on event-related potentials (ERPs) related to emotion, motivational salience, and higher-order cognitive processing (N100, P200 and late positive complex, the LPC), using vocalised non-verbal expressions of sadness (crying) and happiness (laughter) in a 32-participant, within-subject study. Using a repeated measures 2-factor (authenticity, emotion) ANOVA, we show that N100’s amplitude was larger in response to authentic than acted vocalisations, particularly in cries, while P200’s was larger in response to acted vocalisations, particularly in laughs. We suggest these results point to two different mechanisms: (1) a larger N100 in response to authentic vocalisations is consistent with its link to emotional content and arousal (putatively larger amplitude for genuine emotional expressions); (2) a larger P200 in response to acted ones is in line with evidence relating it to motivational salience (putatively larger for ambiguous emotional expressions). Complementarily, a significant main effect of emotion was found on P200 and LPC amplitudes, in that the two were larger for laughs than cries, regardless of authenticity. Overall, we provide the first electroencephalographic examination of authenticity discrimination and propose that authenticity processing of others’ vocalisations is initiated early, along that of their emotional content or category, attesting for its evolutionary relevance for trust and bond formation.

## Introduction

We use cognitive empathy to recognize, understand, and infer others’ states of mind (including emotions, thoughts and intentions), and emotional empathy to share others’ emotions^1–6^. These abilities allowed the evolution of human primates as cooperative species able to form relationships of trust, greatly increasing humans’ survival and reproductive success^7^. Indeed, by allowing the inference of whether to trust another, cognitive empathy makes financial, legal, health, political, and other societal systems, possible. This inference, as well the emotional contagion we receive from others, depends on the perceived authenticity of others’ expressions and intentions.

When produced spontaneously, laughter is usually associated with expressing a positive emotional state and promoting social bonding. However, when acted, it can convey a different social message that can range from positive, to demeaning or aggressive^8,9^. As such, laughter is a powerful tool to influence social group dynamics: it can either blur inter-group boundaries (by welcoming outsiders, through politeness and friction reduction), or to reinforce them (when it is aggressive or ridiculing of outsiders)^9^. Spontaneous crying on the other hand conveys a negative emotional state, intended to evoke urgent help from the listeners or achieve relief^10,11^. However, just as laughter, crying can be produced voluntarily, to induce remorse in face of punishment^12^, or achieve self-beneficial behaviours from others, a strategy used by humans already in infancy^13^. Given that emotional vocalisations can have a myriad of context-dependent social meanings, the ability to discern a genuine from an acted emotional expression is an important first step in the empathic processes of inferring another’s state of mind.

Previous studies have shown that judgements of authenticity in tasks using multi-modal stimuli (e.g., audio-visual, facial and vocal expressions) appear to be driven predominantly by auditory cues^14^, highlighting the importance of studying recognition of authenticity in emotional nonverbal vocalisations in more detail. Within the past decade, we began exploring the processing of affective vocal cues in more depth^15^, particularly vocal displays of emotion such as laughter and crying, with a greater focus on the former^16^. Behavioural evidence indicates that people can distinguish between authentic and acted nonverbal vocalisations with good accuracy^17,18^. Spontaneous, authentic laughter is also rated as more arousing and more positively valenced than its acted counterpart^19^. Further, using fMRI^20,21^, we found that listening to acted (*vs.* authentic) laughter was associated with increased anterior medial prefrontal and cingulate cortical activity (whilst authentic laughter activated the superior temporal gyrus). This brain activation pattern suggests that the processing of acted laughter engages regions typically responsible for higher-order, more deliberate, cognitive skills, such as cognitive empathy, in an attempt to determine the intentions and emotional states behind stimuli that are harder to decode given their ambiguity^21^. Importantly, the above findings strongly suggest that specific cognitive processes and respective brain activation patterns are engaged to decode the (non-)authenticity of non-verbal emotional vocalisations. However, the timing of authenticity discrimination is not yet known. This question can be more suitably examined with electroencephalography (EEG) which allows exploration of the temporal unfolding of cognitive processes with greater temporal resolution than fMRI.

While there are no EEG studies to date that address auditory authenticity processing directly, early ERP components—N100, P200—as well as later—the late positive complex, the LPC—have been proposed to reflect three stages of auditory emotional processing, respectively: sensory processing, integration, and cognitive evaluation^22–25^ (The late positivity observed in response to emotional stimuli is also often referred to as the Late Positive Potential, or LPP, especially in the context of studies using emotional stimuli presented in the visual domain^26^, though also in those using auditory stimuli^27,28^, or those using both audio and visual emotional stimuli^29^. In this paper, we opt for calling the late positivity ERP the LPC, as used in most of the auditory domain literature^22^, for consistency and clarity.). Below, we briefly describe these three stages and associated ERP components to infer the temporal pattern of authenticity recognition and its potential EEG correlates.

During the first stage of auditory emotional processing, basic acoustic cues are extracted from the stimuli, a process reflected in the N100 component. Indeed, EEG studies in general suggest that this component does not differentiate between emotional content^30–34^. Instead, it is modulated by the vocalisations’ acoustic profile^35,36^ and general arousal^37–39^. However, some studies did suggest N100 amplitude can differentiate between emotional and neutral vocalisations^25,40,41^, or between emotional and neutral prosody^42^. Nevertheless, the N100 is thought to reflect predominantly early sensory processing of the stimulus.

During the second stage, auditory cues are integrated, enabling the emotional meaning and salience to be extracted, processes linked to the P200 component^22,23^. Its amplitude appears to differentiate between neutral and emotional stimuli more reliably than N100^31–34,40,43–45^. Crucially, P200 is thought to reflect identification of relevant or salient stimuli in the course of emotional processing, allowing a more in-depth evaluation at later stages^25,33,34,46^. Such motivational salience has been associated with P200 even more specifically in relation to voice qualities (beyond pure emotionality)—such as those indicating the vocalizer’s identity, gender, confidence^47–49^ and even intent, with its amplitude increasing in response to sarcastic *vs.* neutral prosody^50^, and to violation of impressions of fictional characters during reading, as we have shown^51^. As such, P200 is an ERP of potential interest in auditory authenticity discrimination. The motivational salience of acted and authentic emotional vocalisations may well differ as their discrimination is of high evolutionary relevance, by allowing, e.g. the signalling of the need to further evaluate the vocalization’s meaning due to their ambiguity^14,20^ and the avoidance of deception^9,12^.

Finally, the last stage of auditory emotional processing includes a relatively more complex evaluation of the stimuli, including explicit judgements^22,23^. The late ERP component, LPC, is associated with elaborate processing of emotional auditory stimuli^22,25,46,47^. Its amplitude has been shown to increase in response to compliments perceived as sarcastic (insincere, ironic) *vs.* genuine^27,52^, suggesting a possible role in authenticity discrimination in non-verbal vocalizations as well. In line with the implication of higher-order cognition in authenticity recognition are previous fMRI findings demonstrating an involvement of fronto-cortical areas during authenticity discrimination, which were previously linked to mentalizing^20^, a. k. a. cognitive empathy^1–6^.

Since the (non-)authenticity of laughter and crying can convey different social meanings^8,9,12,13,53^, the effect of authenticity may depend on emotion category (laughter *vs.* crying). As mentioned above, the difference between an emotional and a neutral auditory stimulus is generally captured by early components. However, the evidence is less clear as to whether they can differentiate between different emotion ‘categories’. Regarding N100 amplitude, some have reported more negative amplitude for angry *vs.* fearful non-linguistic vocalisations^25^, although others did not find differences between happy *vs.* angry auditory stimuli^40,43^. P200 amplitude has been shown to increase with happy or angry *vs.* sad^45^, and angry *vs.* fearful^25^, with no difference in happy *vs*. angry non-linguistic vocalisations^40^. Regarding valence specifically, a higher amplitude for positive *vs.* negative vocalisations was also demonstrated^54^. Finally, there is also evidence that late components like the LPC can differentiate between six basic emotions in speech prosody, and be independently modulated by arousal^46^, and between anger and sadness, regardless of whether the stimuli is verbal or not^45^, and between crying and laughing in 8-month infants^55^.

In the present study, we characterize, for the first time to our knowledge, the time course of authenticity processing in auditory stimuli (herein, nonverbal vocalisations). Using EEG, we aimed to determine at which stage of emotion processing the distinction between authentic and non-authentic emotional auditory expressions is achieved. In relation to the multi-stage model of emotional processing that distinguishes between sensory processing, integration and cognitive evaluation^22–24^, we hypothesize that authenticity discrimination might begin at the second stage, where the non-authentic sound would be indicated as salient, promoting preferential processing, and carry on to the third, to resolve ambiguity and meaning of the stimuli. As such, we predict that the P200 will increase in response to acted stimuli as opposed to authentic, given its previous implication in motivational salience processing^34,40,45,46^, and the amplitude of the LPC will also increase in response to non-authentic stimuli, given its role in more elaborate processing of social information^27^. Second, since the (non-)authenticity of laughter and crying can convey different social meanings^8,9,12,13,53^, we also aimed to explore whether the effect of authenticity would depend on emotion category (laughter *vs.* crying). Thirdly, to aid the interpretation of our findings, we asked participants to rate vocalisations in terms of their perceived arousal and emotional contagion to explore their association with the ERP amplitudes. We also correlated the amplitudes with vocalisation’s authenticity rating, as well as with authenticity discrimination index that reflects individual’s ability to distinguish between authentic and acted vocalisations^56^.

As authenticity discrimination has been positively correlated with both emotional empathy (ability to share the emotional experiences of others) and cognitive empathy (inferring mental states of others)^56,57^, we explored these traits’ influence on authenticity ratings and EEG measures. Although cognitive empathy is putatively elicited by the authenticity task, given that subjects are asked to infer the authenticity of the vocalizations, the task is bound to implicitly involve emotional empathy as well. Thus, we administered the Empathy Quotient test (EQ^58^; Portuguese translation^59^), which subcomponents tap into emotional and cognitive empathy, and Reading the Mind in the Eyes Test (RMET^60^; Portuguese translation^61^), used as a measure cognitive empathy^62,63^ (often also referred to as Theory of Mind^1–6^). We expected that higher scores in these tests will be associated with better authenticity discrimination and that these measures might correlate with ERP amplitudes associated with processing of authentic and acted vocalisations.

## Results

### Acted vocalisations are rated as less authentic, less contagious, and less arousing

Overall, and as expected, participants rated acted vocalisations as less authentic (*F* (1, 31) = 60.18, *p* < 0.001, *η*_*p*_^2^ = 0.66), less contagious (*F* (1, 26) = 76.05, *p* < 0.001, *η*_*p*_^2^ = 0.75), and less arousing (*F* (1, 24) = 67.69, *p* < 0.001, *η*_*p*_^2^ = 0.74), than authentic vocalisations. They also rated cries as less authentic (*F* (1, 31) = 30.84, *p* < 0.001, *η*_*p*_^2^ = 0.50), less contagious (*F* (1, 26) = 23.76, *p* < 0.001, *η*_*p*_^2^ = 0.48), and less arousing (*F* (1, 24) = 47.58, *p* < 0.001, *η*_*p*_^2^ = 0.66), than laughs (see Table 1 for means and standard deviations). No interactions between the effects of authenticity and emotion were significant. Detailed results are presented in Supplementary Information (Supplementary Text A, Fig. S1) and published in the context of our pupillometry study conducted in the same experimental session and with an 85% sample overlap^64^.

**Table 1:** Summary of the main effects and interactions of authenticity and emotion category on authenticity, emotional contagion, and arousal ratings.

	Omnibus tests of behavioural ratings						
	Effects	Comparison	Mean difference	SD	F (df)	p-value	ηp2
Authenticity	**Authenticity**	**Auth > Act**	**1.14**	**0.83**	**60.18 (31)**	< 0.001*	**0.66**
	**Emotion**	**Laugh > Cry**	**0.91**	**0.93**	**30.84 (31)**	< 0.001*	**0.50**
	Authenticity*Emotion	–	–	–	0.41 (31)	0.525	0.01
Emotional contagion	**Authenticity**	**Auth > Act**	**1.04**	**0.62**	**76.05 (26)**	< 0.001*	**0.75**
	**Emotion**	**Laugh > Cry**	**0.80**	**0.85**	**23.76 (26)**	< 0.001*	**0.48**
	Authenticity*Emotion	–	–	–	3.75 (26)	0.291	0.06
Arousal	**Authenticity**	**Auth > Act**	**1.11**	**0.68**	**67.69 (24)**	< 0.001*	**0.74**
	**Emotion**	**Laugh > Cry**	**1.24**	**0.90**	**47.58 (24)**	< 0.001*	**0.66**
	Authenticity*Emotion	–	–	–	3.49 (24)	0.073	0.13

*Auth* authentic, *Act* acted.

Statistically significant effects (p < 0.05) are signalled with a bold font and an asterisk.

### Early ERPs differentiate between authentic and acted vocalisations

**Figure 1:**
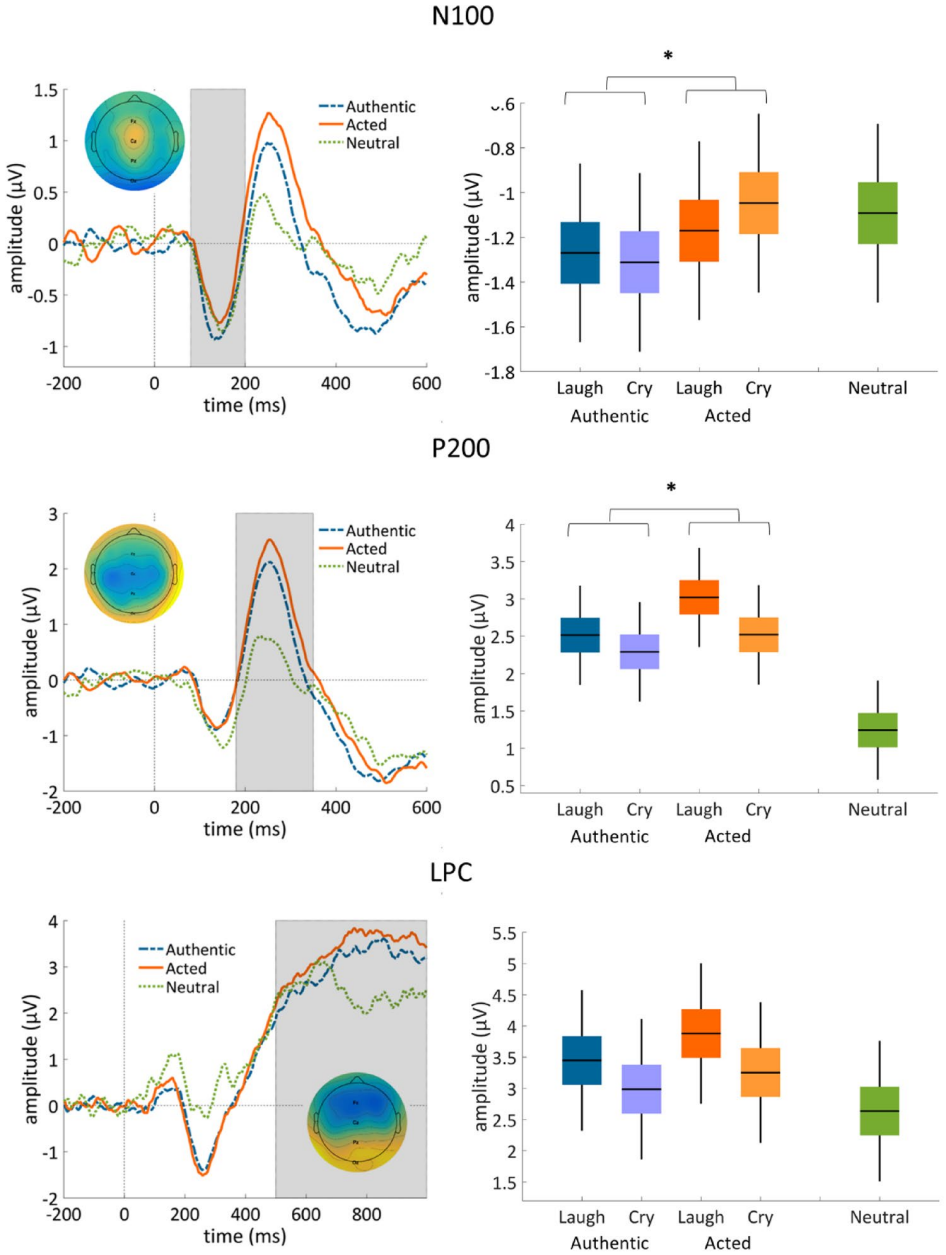
N100 and P200 differentiate between authentic and acted vocalisations. Left panel: grand-average ERP waveforms in response to authentic, acted, and neutral vocalisations for components N100 (80–200 ms; top), P200 (180–350 ms; middle), and LPC (500–1000 ms; bottom), collapsed across laughter and crying. Topographical maps represent the components averaged across the 4 conditions (authentic/acted laughter and crying), within the time windows of interest. Grey-shaded areas represent the analysis time window. Right panel shows box plots of the measured ERP amplitudes. For each box plot, black horizontal line represents the mean, black vertical line is one standard deviation, and coloured patches represent 95% within-subject confidence intervals. Statistically significant effects (Bonferroni-corrected p < 0.05) of authenticity are signalled with an asterisk.

All effects described below are summarized in Table 2 (main effects and interactions) and Table 3 (all pairwise comparisons) and plotted in Fig. 1.

**Table 2:** Summary of the main effects and interactions of authenticity and emotion category on N100, P200 and LPC amplitudes.

	Omnibus tests of ERP amplitudes						
	Effects	Comparison	Mean difference	SD	F (df)	p-value	ηp2
N100	**Authenticity**	**Auth > Act**	**0.182**	**0.43**	**5.67 (31)**	0.024*	**0.16**
	Emotion	–	0.041	0.41	0.32 (31)	0.576	0.01
	Authenticity*Emotion	–	–	–	1.14 (31)	0.244	0.04
P200	**Authenticity**	**Auth < Act**	**− 0.37**	**0.76**	**7.42 (31)**	0.010*	**0.19**
	**Emotion**	**Laugh > Cry**	**0.36**	**0.94**	**4.69 (31)**	0.038*	**0.13**
	Authenticity*Emotion	–	–	–	1.15 (31)	0.291	0.04
LPC	Authenticity	–	0.54	1.29	2.74 (31)	0.108	0.08
	**Emotion**	**Laugh > Cry**	**0.35**	**1.19**	**5.68 (31)**	0.023*	**0.16**
	Authenticity*Emotion	–	–	–	0.11 (31)	0.737	0.004

Statistically significant effects (p < 0.05) are signalled with a bold font and an asterisk.

**Table 3:** Pairwise comparisons between authentic and acted vocalisations in terms of ERP amplitudes (N100, P200, and LPC), separately for laughter and crying.

	Pairwise comparisons of ERP amplitudes							
	Vocalization	Comparison	Mean difference (Auth-Act)	SD	SE	t(df)	p-value	Cohen’s d
N100	Laughter	Auth > Act	+ 0.10	0.61	0.11	− 0.92	0.364	− 0.16
	**Crying**	**Auth > Act**	+**0.27**	**0.56**	**0.10**	− **2.67**	0.012*	− **0.47**
P200	**Laughter**	**Auth < Act**	− **0.51**	**1.28**	**0.23**	− **2.24**	0.03*	− **0.40**
	Crying	Auth < Act	− 0.23	0.77	0.14	− 1.68	0.103	− 0.30
LPC	Laughter	Auth < Act	− 0.43	2.06	0.36	− 1.19	0.244	− 0.21
	Crying	Auth < Act	− 0.27	1.56	0.28	− 0.97	0.341	− 0.17

Statistically significant effects (Bonferroni-corrected p < 0.05) are signalled with a bold font and an asterisk.

### N100

The main effect of authenticity on N100 amplitude was statistically significant (*F* (1, 31) = 5.67, *p* = 0.024, *η*_*p*_^2^ = 0.155), with a more negative N100 amplitude in response to authentic (*M* =  − 1.29, *SD* = 0.69) than acted (*M* =  − 1.18, *SD* = 0.64) vocalisations, irrespective of emotion. The main effect of emotion and the authenticity by emotion interaction were not statistically significant.

Bonferroni-corrected pairwise comparisons showed that this effect was particularly driven by crying, whereby authentic cries (*M* =  − 1.31, *SD* = 0.82) had a more negative N100 amplitude than acted cries (*M* =  − 1.05, *SD* = 0.70, t(31) =  − 2.672, p = 0.012, d = 0.47). When contrasting each authentic to neutral vocalisations, we found no main effect of emotion on N100 amplitude (*F* (2, 62) = 1.82, *p* = 0.171, *η*_*p*_^2^ = 0.055).

### P200

The main effect of authenticity on P200 amplitude was significant (*F* (1, 31) = 7.42, *p* = 0.010, *η*_*p*_^2^ = 0.193), with a more positive amplitude in response to acted (*M* = 2.77, *SD* = 2.20) than authentic (*M* = 2.40, *SD* = 2.33) vocalisations. There was also a main effect of emotion (*F* (1, 31) = 4.69, *p* = 0.038, *η*_*p*_^2^ = 0.131), such that the P200 amplitude was more positive in response to laughter (*M* = 2.76, *SD* = 2.41), than crying (*M* = 2.40, *SD* = 2.15) vocalisations. The 2-way interaction was not significant. Bonferroni-corrected pairwise comparisons showed that this effect was particularly driven by laughter, whereby P200 amplitude was greater for acted laughter (*M* = 3.02, *SD* = 0.44) than to authentic laughter (*M* = 2.51, *SD* = 0.44; t(31) =  − 2.235, p = 0.033, d =  − 0.40).

When contrasting each authentic to neutral vocalisations, we found a main effect of emotion on N200 amplitude (*F* (2, 62) = 20.37, *p* < 0.001, *η*_*p*_^2^ = 0.397). Bonferroni-corrected pairwise comparisons indicated that authentic laughter elicited a grater amplitude (*M* = 2.51, *SD* = 2.49) than neutral (t(31) = 5.12, p < 0.001, d = 0.90), and authentic crying had greater amplitude (*M* = 2.29, *SD* = 2.28) than neutral vocalisations (*M* = 1.24, *SD* = 2.17; t(31) = 5.06, p < 0.001, d = 0.89).

### LPC

We found no statistically significant effect of authenticity on LPC’s amplitude. We found a significant main effect of emotion on mean LPC amplitude (*F* (1, 31) = 5.68, *p* = 0.023*, η*_*p*_^2^ = 0.155), which was more positive in response to laughter (*M* = 3.66, *SD* = 2.49), than crying (*M* = 3.12, *SD* = 1.88) vocalisations. The interaction between authenticity and emotion was not significant.

When contrasting each authentic and neutral vocalisations, we found a main effect of emotion on LPC amplitude (*F* (2, 62) = 3.287, *p* = 0.044, *η*_*p*_^2^ = 0.096). (Only) uncorrected t-tests were significant for authentic laughter (*M* = 3.45, *SD* = 2.78) *vs.* neutral vocalisations (*M* = 2.63, *SD* = 1.84; t(31) = 2.21, p = 0.035, d = 0.39).

### N100 amplitude correlates with ratings of authenticity and arousal

**Figure 2:**
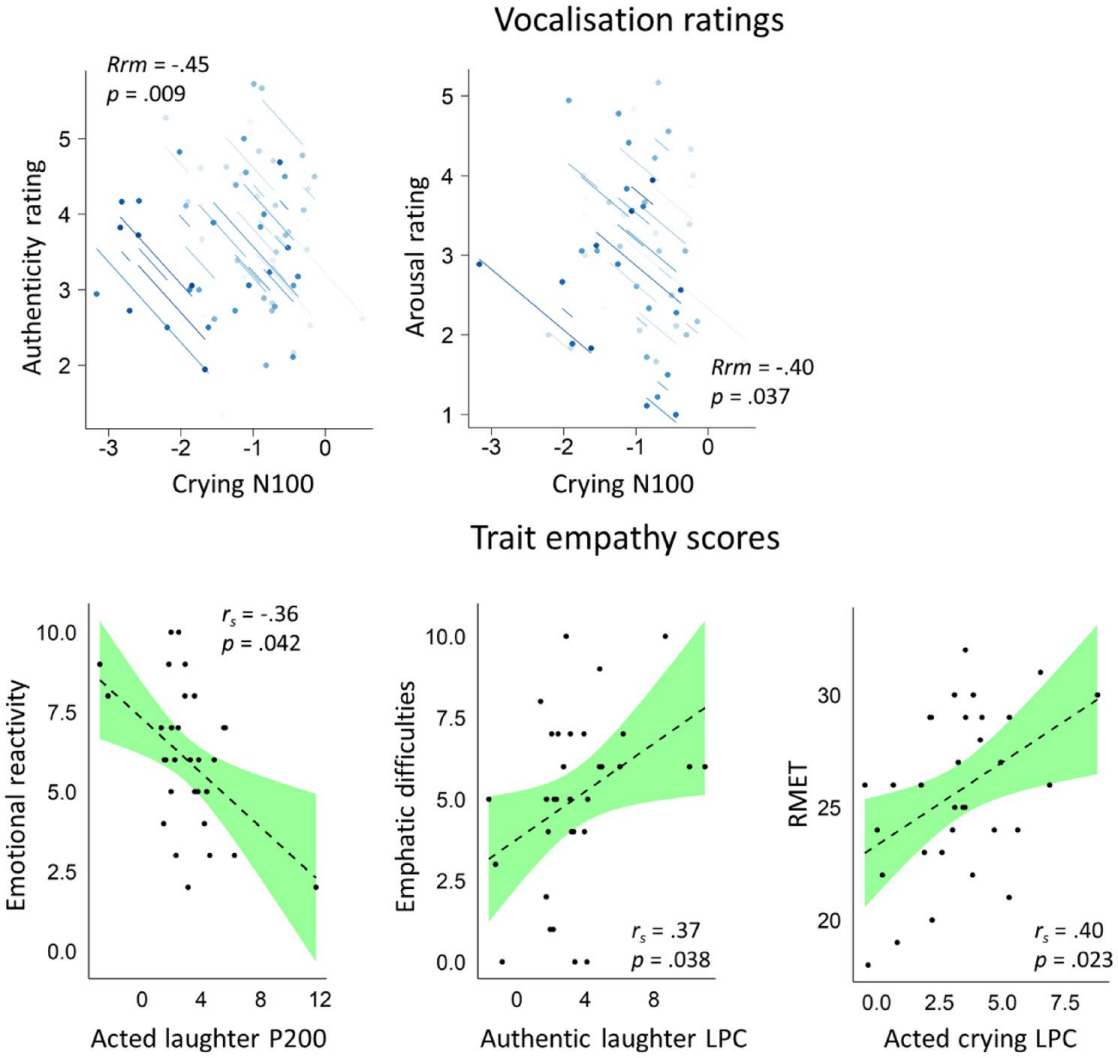
N100 amplitude correlates with ratings of authenticity and arousal, while P200 and LPC amplitudes correlate with trait empathy measures (p < 0.05). Top: visualisation of statistically significant repeated measures correlations between ERP amplitudes and stimulus ratings. Each participant is represented by two points on the graph, corresponding to trial-averaged N100 amplitude/scores in response to authentic and acted crying vocalisations. Bottom: visualisation of significant correlations between ERP amplitudes and trait empathy scores. Green shaded area corresponds to 95% confidence interval. Note that higher score in the emphatic difficulties sub-measure means lower emphatic difficulty.

We found two statistically significant correlations for the N100 component. In particular, N100 amplitude in response to crying correlated with authenticity rating (Rrm =  − 0.45, p = 0.009): the more the crying vocalisations were rated as authentic, the more negative was the associated N100 amplitude. Furthermore, N100 amplitude in response to crying correlated with arousal rating (Rrm =  − 0.40, p = 0.037): the more crying vocalisations were rated as arousing, the more negative the N100 amplitude (see Fig. 2). Full results, including non-statistically significant ones, are provided in Supplementary Information (Table S1).

### P200 and LPC amplitudes correlate with trait empathy measures

We did not find significant correlations between any of the measures and N100. However, the P200 amplitude in response to acted laughter was negatively correlated with a sub-measure of the EQ: emotional reactivity (*r*_*s*_ =  − 0.36, *p* = 0.042). The LPC amplitude in response to authentic laughter was positively correlated with a sub-measure of the EQ: emphatic difficulties (*r*_*s*_ = 0.37, *p* = 0.038), and LPC acted crying amplitude was correlated with RMET (*r*_*s*_ = 0.40, *p* = 0.023; see Fig. 2). The authenticity discrimination index did not correlate with any ERP amplitude or trait empathy scores. Full results, including non-statistically significant ones, are provided in Supplementary Information (Tables S2, S3).

## Discussion

In the present study, we characterize, for the first time to our knowledge, the time course of the processing of vocalisations’ authenticity, using EEG. We focused on three ERP components, two early and one late, during an authenticity recognition task, where subjects were asked to discriminate between authentic and acted vocalisations of laughter and crying. Although we had no expectation of a N100 association with authenticity discrimination (but rather with emotion category), N100 amplitude showed to be under a large main effect of authenticity (authenticity explaining 15.5% of the variance in N100 amplitude unexplained by emotion or its interaction). The amplitude was larger (more negative) for authentic than acted emotional vocalisations, which was especially driven by this amplitude difference in crying. Crucially, as we hypothesized, P200 amplitude, thought to tag motivational significance of stimuli, was larger (more positive; a large effect explaining 19.3% of the amplitude variance unexplained by emotion or its interaction) in response to acted than to authentic vocalisations, a pattern opposite to that of N100. Moreover, this difference appeared to be driven by the amplitude difference in *laughter* (unlike N100).

In detail, while both N100 and P200 amplitudes could dissociate authentic from acted vocalisations, they show an opposite direction of effect, and seemingly a different contrast in relation to neutral vocalisations. For N100, the amplitude was increased (i.e. more negative) for authentic vocalisations compared to acted vocalisations, with the latter seemingly closer in amplitude to the neutral vocalisations. While it is still debated whether N100 responds to emotional content, the auditory N100 is thought to increase in response to arousing stimuli^37–39^. We suggest that higher N100 amplitude for authentic trials in our study might be reflecting a particular sensitivity of this ERP to greater genuineness-derived arousal. In other words, we believe the N100 effect is being driven by the difference in arousal elicited by the authentic vs acted vocalizations, which may contribute to both an emotional empathic reaction as well as to a cognitive empathic decision on the vocalization’s authenticity, downstream. Indeed, in the current experiment, authentic vocalisations were rated as more arousing than acted vocalisations; and both higher authenticity ratings for cries, as well as higher arousal ratings for cries, were associated with an increased N100 amplitude (Fig. 2). Overall, these results suggest that the first cues about a vocalisation’s authenticity may depend on their arousal elicitation and are present even before the categorization of the emotion (as we found no effect of emotion on N100), or of its authenticity, is concluded. Nevertheless, as the ANOVA did not point to a significant difference in N100 between authentic crying and a neutral vocalisation, our interpretation warrants replication and clarification in further studies.

The opposite pattern to N100 was seen for P200. A larger P200 amplitude was elicited by acted *vs.* authentic vocalisations, and by all conditions compared to the neutral vocalisations (the acted laughs being the furthest from neutral, i.e. non-emotional vocalizations). This may suggest that P200 amplitude is particularly triggered by *lack* of authenticity/genuineness (unlike N100). The effect was in the direction we predicted given previous evidence linking increased amplitude to motivational salience, and supporting the P200 amplitude modulation as an early indicator of emotional significance^22,23,25,34,40,46,47^. The P200 effects we observed might thus reflect a higher motivational salience of the acted stimuli^9,53^, serving to signal the need to resolve the expression’s ambiguity and the intention of the speaker, while authentic emotions require less effort to decipher. This echoes an interpretation previously offered in the fMRI literature^20^. In this light, the ambiguity of the expression brings a need for the listener to allocate additional resources to resolve it—and ascertain the adequate level of trust. It is not clear what aspects of the non-authentic stimuli make it salient by itself. One possibility is that acted vocalisations might violate our internal template of authentic displays of emotion, and thus authenticity recognition might operate on the basis of mismatch or incongruence detection, bringing it conceptually closer to studies investigating processing of emotionally ambiguous stimuli (such as pictures of faces with angry eyes and happy smiles^65^. As the design of the current experiment does not allow one to dissociate processing of authenticity from mismatch detection, nor to isolate what aspect of the stimuli is “salient”, future studies might introduce conditions that directly modulate congruency and salience independently from authenticity to narrow down the exact mechanism through which individuals are able to make accurate authenticity judgments.

Importantly, when we explored the simple effects of authenticity on ERP amplitudes for laughter and crying separately, we observed that the main effect on N100 was driven by the simple effect in crying, and the one on P200 by the simple effect in laughter (both effects being statistically significant). This evidence converges with the pupillometry evidence we have recently published from roughly the same sample of participants^64^, collected during the same experimental session. Therein, we observed a similar pattern of difference between laughter and crying. In that study, there was a negative effect of authenticity on pupil dilation in laughter (i.e. pupil size larger for acted than authentic laughter) whilst a positive one in crying (i.e. pupil size larger for authentic than acted crying). We interpreted this pattern as indication that authenticity discrimination in laughter is driven by relatively higher-order cognitive processing, while in crying it relies on a relatively more automatic arousal response. Acted (vs. authentic) laughs trigger a high motivational salience leading to a drive to decipher the other’s intention, whilst authentic (vs. acted) cries trigger an immediate high-arousal response leading to a drive to act to solve a potentially threatening situation. The present EEG data concurs with that interpretation, since arousal-related N100 was mostly driven by the authentic-acted difference in cries; and the P200 (linked to early tagging of motivational salience/significance, a first step towards more elaborate processing) was driven by the authentic-acted difference in laughter.

Complementarily, in terms of emotion, N100 did not differentiate between laughter and crying, which adds to the so far inconsistent evidence in the literature that this early component is sensitive to emotion category and in what direction^25,40,45^. For P200, and although its link to emotion categorisation is also still debated^66^, we did find laughter to elicit higher amplitudes than crying, in line with another study using laughter and crying vocalisations^54^. In the present study, emotion explained 13.1% of the otherwise unexplained amplitude variance (a quasi-large effect). Additionally, P200 amplitudes in response to authentic laughter and crying very significantly larger than to neutral vocalisations. Regarding the late component (LPC), although we expected its amplitude to be sensitive to both emotion authenticity and category, we found only the latter (LPC amplitude being larger for laughter than for crying), explaining a large portion, 15.6%, of the amplitude variance left unexplained otherwise. When we compared LPC amplitudes in response only to authentic vocalisations *vs.* neutral, the main effect of emotion was detected, although pairwise comparisons did not point to a specific condition (laughter, crying, or neutral) that was driving this effect.

Finally, to try to further constrain the interpretation of the results, we explored whether ERP amplitudes were correlated with any trait empathy scores measured in this experiment. To that end, we found that P200 in acted laughter was associated with the emotional empathy sub-measure of the EQ: emotional reactivity. Expectedly, the correlation was negative, i.e., the higher the emotional reactivity trait of the subjects, the smaller the P200 amplitude in response to acted laughter. Speculatively, this could mean that individuals with higher emotional empathy might be able to recognise non-authentic laughter with less neuronal resources, reflected in a lower P200 amplitude. Regarding the LPC, lower empathic difficulty (reflected by a higher score in the emphatic difficulties sub-measure of the EQ) was associated with greater LPC amplitude in response to authentic laughter. Furthermore, a higher score in the RMET (meant to measure cognitive empathy) was associated with greater LPC amplitude during acted crying. We report these effects for completeness—and as they may tentatively suggest, expectedly, that LPC amplitude changes are particularly influenced by cognitive empathy skills. Nevertheless, we think they do not aid the interpretation of our LPC findings.

### Potential limitations

Since early ERP components, including N100 and P200, are sensitive to low-level acoustic properties^36^, authenticity discrimination is likely to be driven by the different acoustic properties of authentic *vs.* acted vocalisations^18,19^. Nevertheless, in the present dataset, complementary analyses showed that the average amplitude of neither ERP component was correlated with intensity, mean pitch (fundamental frequency), intensity, or duration of the vocal stimuli. Hence, we suggest that the specific P200 and N100 amplitude effects we found may not be directly attributed to the low-level factors we tested.

In regards to the LPC, the lack of a significant difference in amplitude between authentic and acted vocalisations conflicted with our initial expectation, and with studies suggesting the role of mentalising, and thus cognitive empathy, in authenticity processing^20^—which is usually associated with modulation of late ERPs^67^. However, visual inspection of the plotted LPC amplitudes (see Fig. 1) between authentic and acted vocalisations does suggest a trend. Furthermore, as noted above, the duration of the vocalisations ranged, on average, from 2182 to 2685 s (see Supplementary Information: Table S4). In contrast with a visual stimulus, a vocalisation is not available in its entirety at once, but is unfolded continuously over the presentation time. Therefore, it is possible that the differences in the LPC amplitude could arise later than the analysis time window we selected a priori (500–1000 ms). Another related possibility is that the task did not require participants to decipher the *meaning* behind the vocalisations; thus, the more elaborate processing typically related to LPC might not have been induced by our design, albeit it might have been sufficient in abovementioned MRI context. Furthermore, the fact that the failure to discriminate acted from authentic did not carry consequences for the participants may also have led to a weaker involvement of the LPC. To address this, a punishment/reward aspect to authenticity discrimination may be useful in future studies. Finally, in contrast to a previous study^56^, we did not find significant correlations between authenticity discrimination index and trait empathy scores. We speculate that this might be due to a small sample used here, in comparison with 119 participants included in that study, and the use of different empathy measures.

Finally, given that we have used the original stimuli validated^68^ and employed in several previous studies of authenticity discrimination^20,69^—to aid literature comparability—the stimuli are of different length (which comes with their intrinsic ecological validity). In the ERP analysis, this should not to be problematic, given that the latest time window is under 1000 ms, while none of the stimuli had shorter duration than that. However, as the behavioural ratings are based on the full stimulus length, these cannot be fully relatable with ERP results.

## Conclusion and further research

Together, these results suggest that the processing of authenticity in vocalised emotions is detected rapidly (as shown by its modulation of N100 and P200 amplitudes), while a later component’s (LPC, linked to more deliberate, cognitive, evaluation) engagement was only tentative. Given previous evidence, we suggest that N100 and P200 engagement may be due to them reflecting arousal and motivational salience attribution, respectively. Early processing of authenticity may be relevant for trust bond formation, protection from deceit and survival in a social context. Therefore, is conceivable that authenticity recognition mechanisms are built on top of the existing, general salience detectors that allow us to pick-up on important information in the environment. Relating our results to the multi-stage model of emotion processing^22–24^, we propose that authenticity discrimination is carried on during the second, integration stage (as revealed by the P200 in this study), although differences in the arousal level between authentic and acted vocalisations might already mark its impact during the sensory processing stage (as revealed by N100 here).

Still, what drives salience attribution to non-authentic emotional vocalisations in the first place needs to be further narrowed down. We also suggest that what drives the early stages of authenticity recognition in crying and laughter might not be the same—with the former achieved through arousal, and the latter through a higher-order cognitive processing, en par with our pupillometry findings^64^. In sum, the current study—being the first to investigate authenticity recognition using EEG—hopefully serves as a driver of new hypotheses and independent studies—which will be helpful to substantiate the novel findings presented here.

## Materials and methods

### Participants

A total of 38 participants participated in the experiment, recruited through the lab’s online recruitment platform and social media. The inclusion criteria were right-handedness (assessed with Edinburgh Handedness Inventory^70^, 20–30 years of age, European Portuguese as a first language, and no past or current psychiatric illness, no psychotropic medication use, and no history of drug addiction or current consumption in the last 6 months. Additionally, women had to be on the active-intake weeks of contraceptive pills, as previous research suggested that affective task performance varies according to the menstrual cycle^71^. The study was approved by the Ethics Committee of the Medical Academic Centre of Lisbon (Centro Académico Médico de Lisboa) and all volunteers signed an informed consent form and were paid for their time. The study has been performed in accordance with the Declaration of Helsinki.

As six participants were excluded due to technical problems and/or errors in data acquisition (e.g. EEG markers not set properly), data from 32 participants (16 men and 16 women; age range 21 to 28 years old; *M* = 23.4, *SD* = 1.65) was analysed. To characterize inter-subject variability in mood, working memory and psychopathology which can affect task compliance and performance—and potentially identify outliers to discard from analysis—we administered standard questionnaires/test. No participants were excluded based on these (see Supplementary Information: Supplementary Text B for questionnaires list, results, and justification). To assess cognitive and emotional empathy, we collected the Empathy Quotient (EQ; 22-item version: Wakabayashi et al*.*^72^; Portuguese version: Rodrigues et al*.*^59^) (*M* = 21.8, *SD* = 7.80), the Reading the Mind in the Eyes Test (RMET: Baron-Cohen et al*.*, 2001; Portuguese version: Mouga and Tavares^61^) (*M* = 25.7, *SD* = 3.59).

### Stimuli

The emotional stimuli (laughter, crying, and neutral vocalisations) were developed at the University College of London^68^ and have been used in previous behavioural and neuroimaging studies we conducted^20,69^. Authentic vocalisations consisted of spontaneously produced vocalisations either in response to a humorous video (authentic laughter) or recalling of truly upsetting events (authentic crying). Acted vocalisations were acted expressions under full voluntary control. 16-bit, mono .wav files were created, sampled at 44.1 kHz. The audio was normalized for the root-mean-square (RMS) amplitude using Praat software^74^. The auditory stimuli were presented binaurally through a set of Sennheiser CX 3.00 ear-canal phones at a comfortable listening level that was individually adjusted at the start of the experiment. Given that auditory ERP components like N100 and P200 are sensitive to changes in the stimuli’s low-level acoustic properties, and such properties mediate recognition of vocalisations’ authenticity^17,46^, we also extracted acoustic properties in an attempt to consolidate this evidence. We extracted duration (ms), mean fundamental frequency (F(0), which is perceived as pitch), and mean intensity (dB), using the Praat software. We later found no significant correlations between acoustic properties and ERP peak amplitudes; detailed results are presented in Supplementary Information (Supplementary Text C); as such, we can consider the forthcoming ERP waveforms as not driven predominantly by the low-level acoustic properties we tested.

### Procedure

The experiment consisted of one single session (lasting 2.5 h), divided in two tasks: (1) the EEG-recorded authenticity task, and (2) the non-EEG-recorded arousal and contagion rating task. After EEG setup, participants were taken to a quiet room, seated 80 cm away from the monitor and instructed to remain as still as possible. The experiment was developed and presented using Psychtoolbox 3^75^ for MATLAB version 8.3.0 (R2014a). In all tasks, participants were asked to evaluate emotional vocalisations on a 7-point Likert scales, using a response pad, as intuitively as possible. Buttons of the response pad were marked with the Likert scale numbers (left hand—1, 2, 3; right hand—4, 5, 6, 7). Given the long duration of the task (36 min), three pauses of 30 s were distributed equally throughout the experiment for the participant to rest, to minimise fatigue. Pupillometry data were recorded alongside the EEG and are reported elsewhere^64^.

### Authenticity task

**Figure 3:**
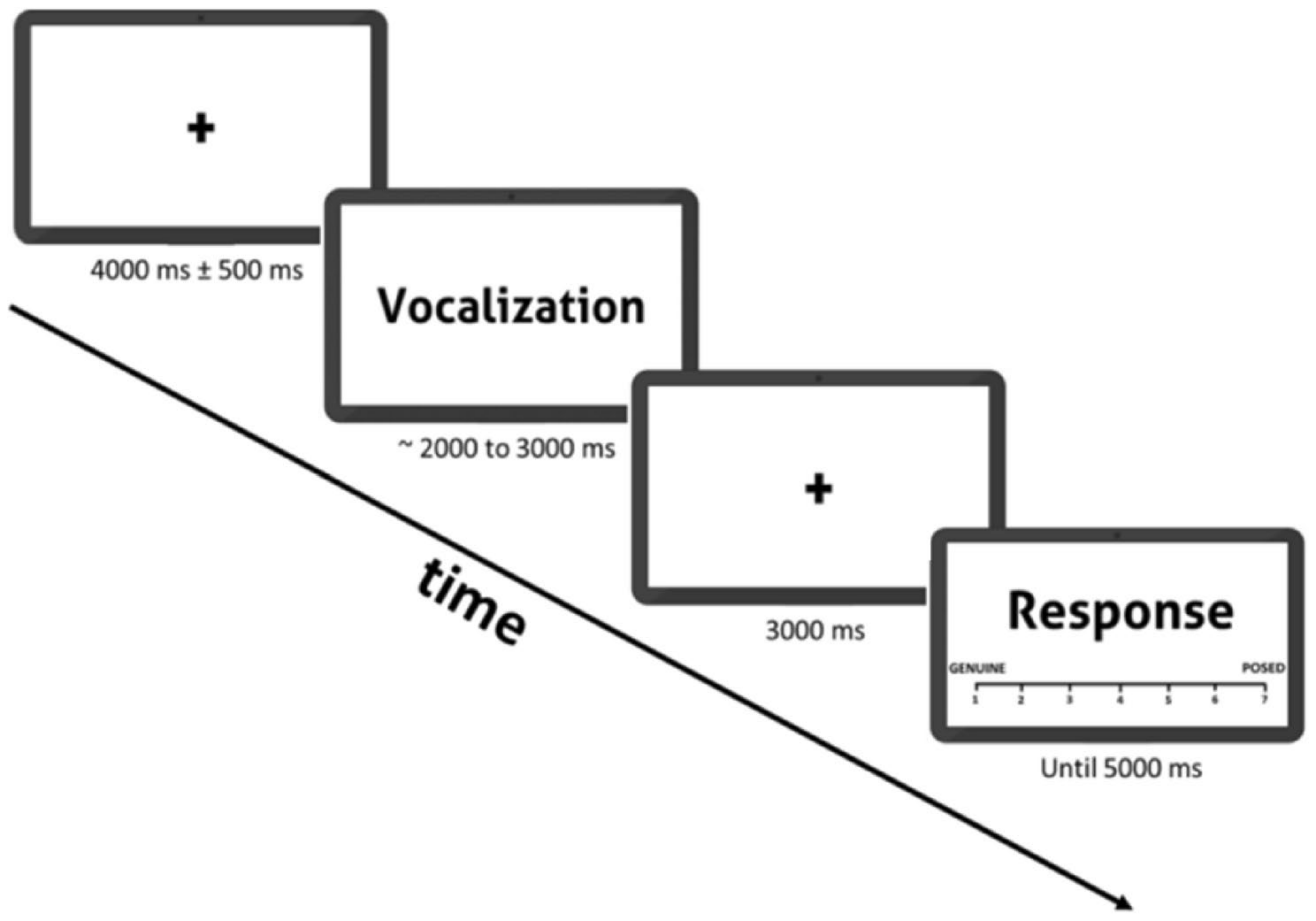
Outline of a single experimental trial in the authenticity task.

Before starting the authenticity task, participants were told that they would hear a set of emotional vocalisations that they would rate in terms of their authenticity (authentic *vs.* acted), as well as a set of neutral sounds that they should attend to, but not rate. A trial started with a 4000 ms fixation cross with a jitter of 500 ms, followed by the presentation of each stimulus. After presentation, and after a 3000 ms interval, a rating screen appeared, and participants had up to 5000 ms to rate the previously presented stimulus. Participants used a 7-point Likert scale to rate the perceived authenticity of the stimulus, ranging from 1 (“Genuine”—authentic), to 7 (“Posed”—acted). The stimuli sequence was pseudo-randomized and fixed for all participants, in a way to ensure that the possible transitions from one condition to another were distributed equally throughout the task. A total of 72 unique emotional vocalisations were used in the experiment (18 for each condition: spontaneous laughter, acted laughter, spontaneous crying, and acted crying). Each emotional vocalisation was presented twice, and thus participants listened to 144 emotional vocalisations in total (36 per condition). As control conditions, additional 30 neutral vocalisations (i.e. vowel ‘ah’ produced with a neutral intonation) were presented. Thus, a grand total of 204 trials were presented in the EEG authenticity task. The entire task lasted around 36 min. The experimental design is outlined in Fig. 3.

### Arousal and emotional contagion ratings

After EEG data acquisition, participants were instructed to evaluate the perceived arousal and emotional contagion of the previously presented vocal stimuli in a 7-point Likert scale (Arousal: 1—Low arousal, 7—High arousal; Emotional Contagion: 1—Not contagious at all, 7—Highly contagious). In the first block of stimuli, participants were asked to rate the perceived arousal of each stimulus, whereas in the second block they were asked to rate the perceived emotional contagion of the same stimulus. Each block had a total of 72 trials (with the same 18 spontaneous laughter, 18 acted laughter, 18 spontaneous crying, 18 acted crying vocalisations). A trial had the following sequence: a fixation cross presented during 1500 ms with a jitter of 500 ms, presentation of the vocalisation, fixation cross during 1000 ms, and lastly, perceived arousal or emotional contagion rating depending on the block. The task was presented in a fixed sequence which accounted for the number of each transition type and had a total of 144 trials (15 min). Each vocalisation was only presented once in each block.

### EEG acquisition and preprocessing

EEG was recorded using a 64-channel Brain Vision actiCHamp system (Brain Products, Munschen, Germany) at a sampling rate of 512 Hz with two reference electrodes placed on the left and right mastoids. Bipolar horizontal and vertical electro-oculograms were acquired through 4 flat-type facial electrodes: two electrodes were placed at the outer corner of each eye (horizontal electro-oculogram) and two electrodes were placed below and above the left eye (vertical electro-oculogram). Electrode impedance was kept under 10 kΩ for all electrodes. The data was preprocessed offline using Brain Vision Analyser software (Brain Products, GmbH, Munich, Germany), EEGLAB^76^ and custom functions (the latter two written for Matlab, Mathworks, Natick, Massachusetts). The data was band-pass filtered offline between 0.1 and 30 Hz using zero phase shift IIR Butterworth filters, with an additional 50 Hz notch filter, and re-referenced to average (after removal of noisy electrodes). The data was time-locked to the onset of vocalisations and segmented into epochs (− 200 to 1000 ms). Epochs with non-stereotypical artifacts (large muscle artifacts, singular events) were manually removed. On average, 6% of trials were removed (most participants had a removal rate ranging 0% to 13%, and one had 28% trials removed). The epochs were further cleaned from ocular artifacts using Independent Component Analysis (ICA; infomax restricted algorithm). An ocular electrode was entered into the ICA to flag components related to ocular activity on the basis of sum of squared correlations with the vertical and horizontal electrodes. In case the ocular electrodes were too noisy, a clean frontal electrode with clear ocular activity was used instead. Removed electrodes were reconstructed using spline interpolation. Pupillometry data was also concomitantly collected, for which results have been reported elsewhere^64^.

### ERP analysis

The time intervals and electrodes subjected to statistical analysis were selected on the basis of subject-averaged ERP waveforms and topographic maps, collapsed across all experimental conditions to avoid bias^77^. Electrode sites with the highest activity within the selected time window were chosen. Details and plots used to make these decisions are provided in Supplementary Information (Supplementary Text D, Fig. S4). The electrode clusters and time-windows for each component were as follows: (1) N100: 80–200 ms, electrodes: C1, C2, C3, C4, Cz, CP1, CP2, CP3 and CPz; (2) P200: 180–350 ms, electrodes Cz and FCz; and (3) LPC: 500–1000 ms, electrodes: PO3, PO4, PO7, PO8, POz, O1, O2, and Oz. To increase precision of the measurement, the mean N100 and P200 amplitudes were measured between the peaks’ onset and offset^78,79^. Further details are presented in Supplementary Text D.

### Effect of authenticity and emotion on ERP and vocalisation ratings

We used a series of 2-way ANOVAs to estimate the main and interaction effects of the within-subject independent variables emotion (laughter, crying) and authenticity (authentic, acted) on the extracted amplitude peaks of each ERP component separately (N100, P200 and LPC), using SPSS (version 25, SPSS Inc., Chicago, IL, USA). We did not have specific hypotheses in regard to components’ latencies but provide the analysis in Supplementary Information (Table S7), to inform further research. Since there was no equivalent “neutral” condition to acted vocalisations as there was for authentic ones, this condition could not be included in the model. Nevertheless, to aid interpretation of results, we ran a separate 1-way ANOVA to estimate differences between authentic laughter, authentic crying, and neutral vocalisations, per ERP component. To estimate the main and interaction effects of emotion and authenticity on the vocalisation ratings (authenticity, arousal, and contagion ratings), we applied the above-mentioned 2-way ANOVA design. To make the interpretation of the authenticity rating more intuitive, we reversed it so that higher authenticity scores meant that vocalisation was perceived as more authentic. We considered an effect statistically significant when its test-statistic p-value was below 0.05. We followed main effects and interactions with pairwise post-hoc tests (Bonferroni-corrected for multiple comparisons). As the ANOVA effect size measure, we used partial eta squared (ηp2), and considered the following standard ranges: below 0.01 as marginal, 0.01–0.06 as small, 0.06–0.14 as medium, and above 0.14 as large effect sizes^80,81^; in post-hoc comparisons, we report Cohen’s d. Error bars used in all plots are 95% within-subject confidence intervals^82,83^. All box plots were generated using a notBoxPlot Matlab function^84^, modified to incorporate the within-subject confidence intervals.

### Correlation between ERPs and vocalisation ratings, trait empathy scores and the authenticity discrimination index

To explore associations between ERP amplitudes and vocalisations ratings, in each emotion separately, we used a repeated measures correlation (rmcorr package in RStudio software, version 1.0.143; Bakdash and Marusich^85^; R Core Team^86^). Since each unique stimulus was presented twice, we considered only the ratings made after the first stimulus presentation to capture the initial authenticity perception. Furthermore, we used Spearman’s rank correlation to explore correlations between the same ERP measures and trait empathy scores (as measured by EQ and RMET) and the authenticity discrimination index. The discrimination index refers to the individual’s ability to determine the authenticity of the stimulus and is computed by subtracting the average authenticity ratings of acted stimuli from the average authenticity ratings of authentic stimuli^56^. We also tested for a correlation between this index and the abovementioned empathy scores. As these complementary analyses were ran with the sole purpose of aiding the interpretation of the main findings (see “Materials and methods” section above), we have not performed multiple-comparisons correction on these^87^, and the corresponding statistically significant results (p < 0.05) should be regarded as suggestive.

## Supplementary Information

Supplementary Information.

## Data Availability

Data and code used in analyses are made available on the Open Science Framework database (https://osf.io/rudt5/?view_only=976b60ac3b134b899859ecda493dd2cd).
