# Author Correction: The neural basis of authenticity recognition in laughter and crying

**DOI:** 10.1038/s41598-023-28360-2

**Published:** 2023-01-25

**Authors:** Maciej Kosilo, Mónica Costa, Helen E. Nuttall, Hugo Ferreira, Sophie Scott, Sofia Menéres, José Pestana, Rita Jerónimo, Diana Prata

**Affiliations:** 1 Instituto de Biofísica e Engenharia Biomédica, Faculdade de Ciências da Universidade de Lisboa, Campo Grande 016, 1749-016 Lisbon, Portugal; 2 William James Center for Research, Lisbon, Portugal; 3 Department of Psychology, Lancaster University, Lancaster, UK; 4 Institute of Cognitive Neuroscience, University College London, London, UK; 5 APPsyCI - Applied Psychology Research Center Capabilities & Inclusion, ISPA - Instituto Universitário, Lisbon, Portugal; 6 Departamento de Biociências, ISPA – Instituto Universitário, Lisbon, Portugal; 7 Instituto Universitário de Lisboa (Iscte-IUL), CIS-Iscte, Lisbon, Portugal; 8 Department of Neuroimaging, Institute of Psychiatry, Psychology and Neuroscience, London, UK

Correction to:* Scientific Reports* 10.1038/s41598-021-03131-z, published on 9 December 2021

The original version of this Article contained an error in Table 3, column “Comparison”, where the greater-than and less-than signs in rows “N100” and “P200” were incorrect. Additionally, to increase the readability of the Table, data in row “LPC” was adjusted; values “Acted” and “Authentic” were abbreviated to “Act” and “Auth”; the asterisks present in rows “N100: Crying” and “P200: Laughter” were deleted to avoid redundancy; and the negative symbols present in column “Mean difference (authentic acted)” were changed to positive symbols. The correct and incorrect Tables appear below.

Incorrect:

**Table:** 

	Vocalization	Comparison	Mean difference (authentic acted)
N100	Laughter	Acted > Authentic	− 0.10
	**Crying**	Acted > Authentic*	− **0.27**
P200	**Laughter**	Authentic > Acted*	**− 0.51**
	Crying	Authentic > Acted	− 0.23
LPC	Laughter	Acted > Authentic	− 0.43
	Crying	Acted > Authentic	− 0.27

Correct:

**Table:** 

	Vocalization	Comparison	Mean difference (Auth-Act)
N100	Laughter	Auth > Act	+ 0.10
	**Crying**	**Auth > Act**	+ **0.27**
P200	**Laughter**	**Auth < Act**	**− 0.51**
	Crying	Auth < Act	− 0.23
LPC	Laughter	Auth < Act	− 0.43
	Crying	Auth < Act	− 0.27

The original Article has been corrected.

