# Supplementary material for: The neural basis of authenticity recognition in laughter and crying
Source: Sci Rep. 2021 Dec 9;11:23750. doi: 10.1038/s41598-021-03131-z (PMC8660868; doi:10.1038/s41598-021-03131-z)
Supplement: Supplementary file 1 — Supplementary Information. [file 41598_2021_3131_MOESM1_ESM.docx]

Supplementary Figures


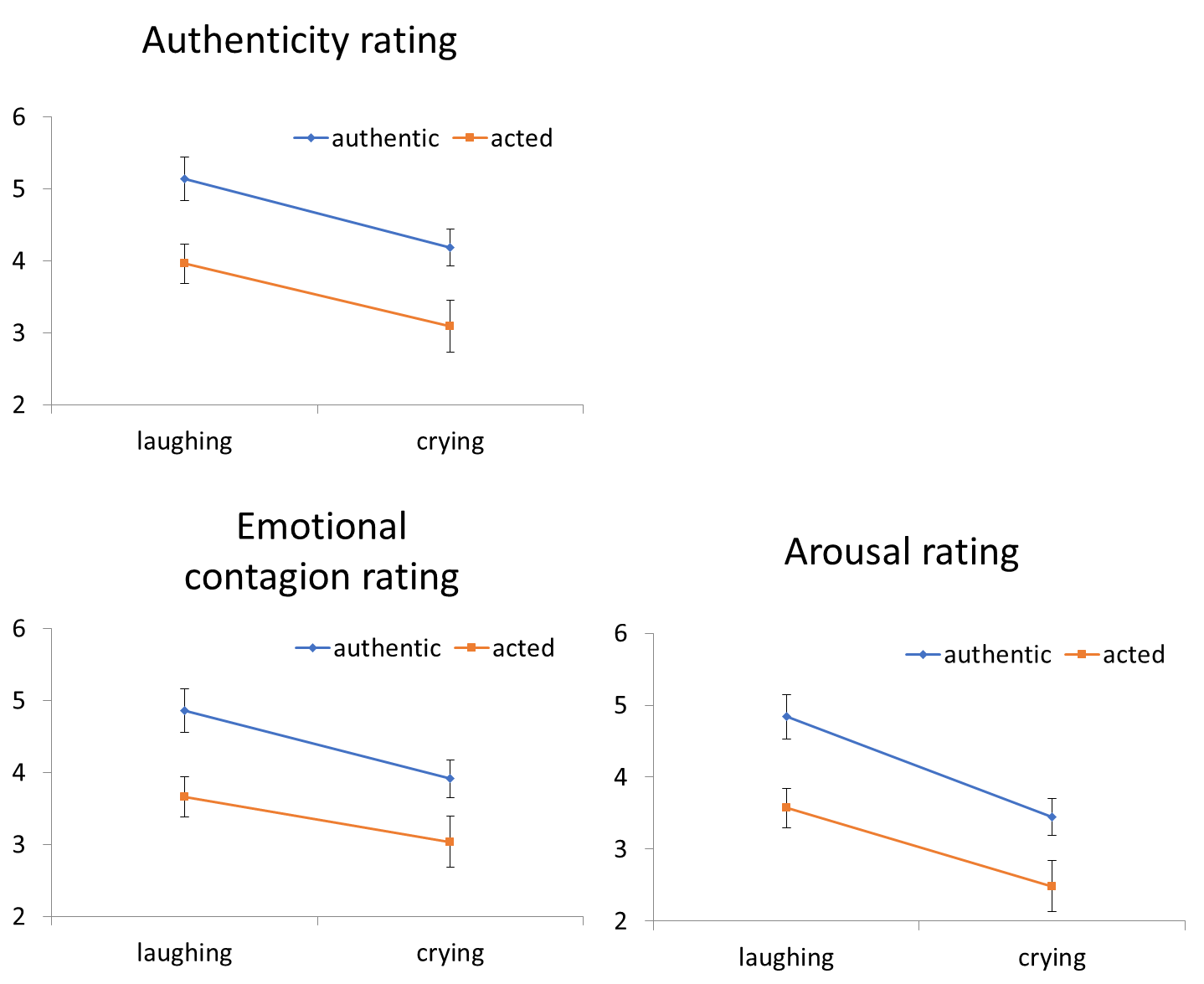


Fig. S1. Average authenticity, emotional contagion, and arousal ratings. Higher rating meant that vocalization was perceived as more authentic, contagious, or arousing. Error bars are 95% within-subject confidence intervals.


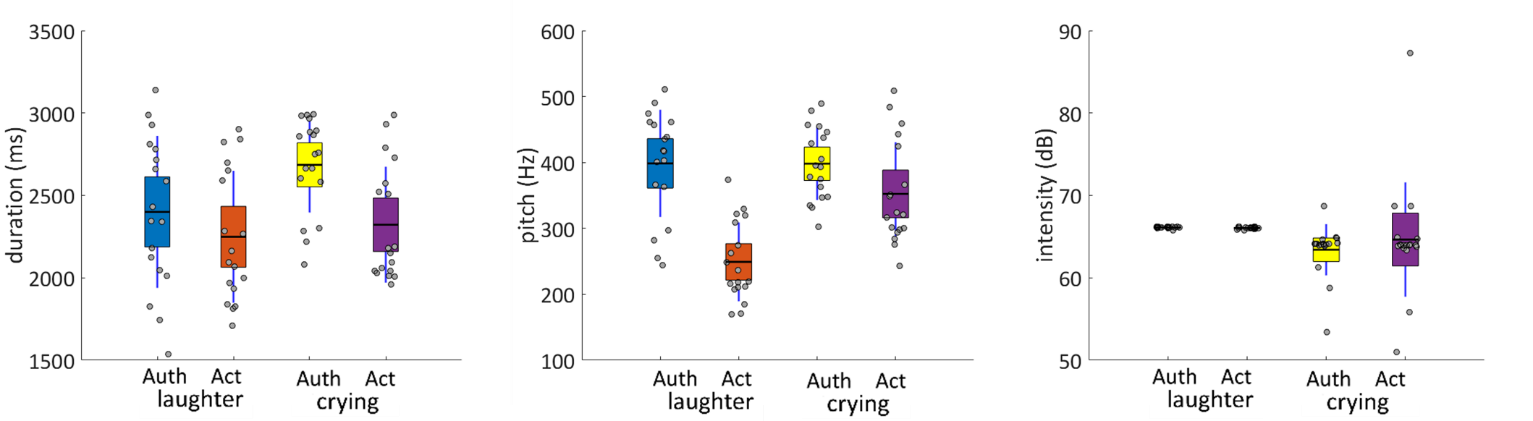


Fig. S2. Comparison of mean acoustic properties (duration, pitch, and intensity) across experimental conditions: authentic and acted laughing, and authentic and acted crying. The coloured patches represent 95% confidence interval, the blue lines are one standard deviation, the black line represents the mean, and the points represent individual value for each stimuli in the condition (18 per condition).


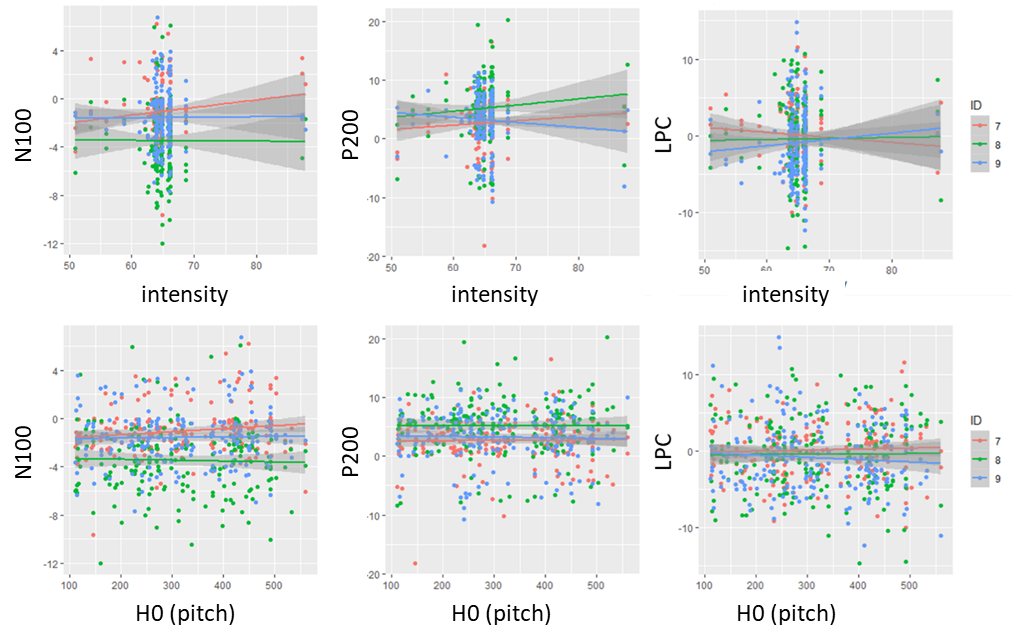


Fig. S3. Association between ERP components N100 (Left), P200 (middle) and LPC (right) and auditory stimuli intensity (upper row) and pitch (bottom row). Each color represents one trial of one participant, solid lines are model fits, and shaded areas represent 95% confidence interval. Trials from all conditions and all participants were included in the analysis, but only three participants were selected for demonstration purposes in the above graph.


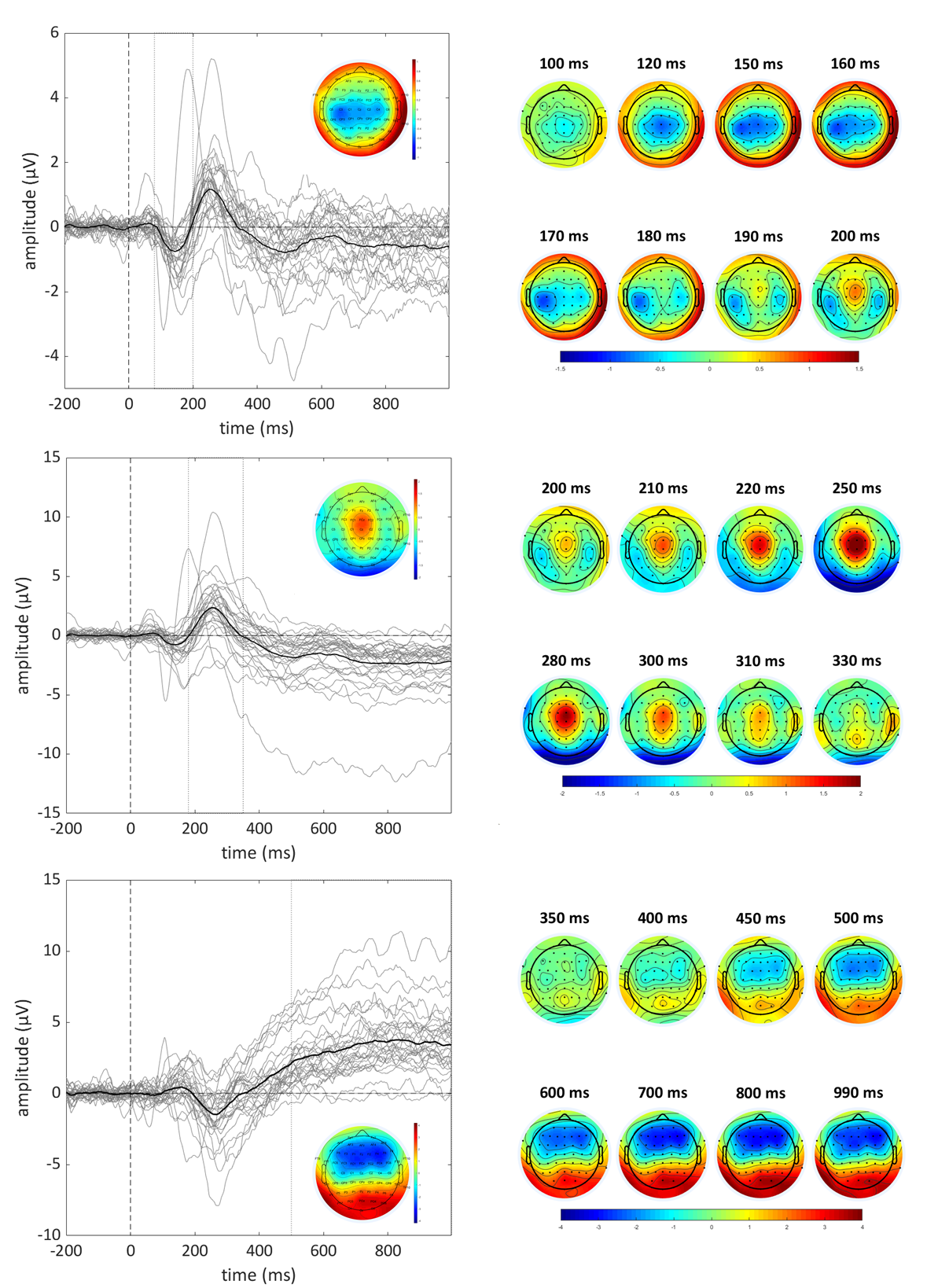


Fig. S4. Left panels: grand-averaged ERP waveforms (black lines) presented against single-subject ERPs (gray lines) for components N100 (top), P200 (middle) and LPC (bottom). The N100 reflects averaged activity at electrodes C1, C2, C3, C4, Cz, CP1, CP2, CP3 and CPz, the P200 corresponds to electrodes Cz and FCz. and LPC shows activity at electrodes PO3, PO4, PO7, PO8, POz, O1, O2, and Oz. Topography inserts depict average activity in the time window of interest, averaged across all experimental conditions (80-200ms for N100, 180-350ms for P200, and 500-1000ms for the LPC). Right-side of the panel shows topography at different latencies, around time-windows of interest. The final time-window of interest as well as electrodes entered into statistical analysis was chosen based on these topographies, along with the inspection of single-subject ERPs.

Supplementary Tables

**Table S1.** Repeated measure correlation coefficients (Rrm) between ERP amplitudes and authenticity, emotional contagion, and arousal ratings, and their respective p-values and confidence intervals. Each participant is represented by two data points, corresponding to trial-averaged ERP amplitude/scores in response to authentic and acted vocalisations. Statistically significant effects (p <.05) are underlined and signaled with an asterisk.

| **Authenticity rating** | | | | | | |
| --- | --- | --- | --- | --- | --- | --- |
|  |  | Rrm | df | p-value | 95% CI | |
| N100 | **Laughter** | 0.08 | 31 | 0.647 | -0.28 | 0.43 |
|  | **Crying** | **0.45** | **31** | **0.009*** | **0.11** | **0.69** |
| P200 | **Laughter** | 0.12 | 31 | 0.498 | -0.24 | 0.46 |
|  | **Crying** | 0.29 | 31 | 0.106 | -0.08 | 0.58 |
| LPC | **Laughter** | 0.16 | 31 | 0.378 | -0.21 | 0.49 |
|  | **Crying** | 0.24 | 31 | 0.178 | -0.13 | 0.55 |
| **Emotional Contagion rating** | | | | | | |
|  |  | Rrm | df | p-value | 95% CI | |
| N100 | **Laughter** | -0.06 | 27 | 0.738 | -0.43 | 0.32 |
|  | **Crying** | -0.25 | 27 | 0.184 | -0.58 | 0.14 |
| P200 | **Laughter** | -0.18 | 27 | 0.35 | -0.52 | 0.21 |
|  | **Crying** | -0.21 | 27 | 0.278 | -0.55 | 0.19 |
| LPC | **Laughter** | -0.21 | 27 | 0.284 | -0.54 | 0.19 |
|  | **Crying** | -0.13 | 27 | 0.513 | -0.48 | 0.27 |
| **Arousal rating** | | | | | | |
|  |  | Rrm | df | p-value | 95% CI | |
| N100 | **Laughter** | -0.11 | 25 | 0.574 | -0.49 | 0.30 |
|  | **Crying** | **-0.40** | **25** | **0.037*** | **-0.69** | **-0.01** |
| P200 | **Laughter** | -0.33 | 25 | 0.091 | -0.64 | 0.07 |
|  | **Crying** | -0.29 | 25 | 0.137 | -0.62 | 0.12 |
| LPC | **Laughter** | -0.20 | 25 | 0.323 | -0.55 | 0.21 |
|  | **Crying** | -0.06 | 25 | 0.75 | -0.45 | 0.34 |

**Table S2.** Correlations between ERP amplitudes, trait empathy scores, and authenticity discrimination index. Statistically significant effects (p <.05) are underlined and signaled with an asterisk.

|  | **N100** | | | | | | | |
| --- | --- | --- | --- | --- | --- | --- | --- | --- |
|  | **Authentic laughter** | | **Acted laughter** | | **Authentic crying** | | **Acted crying** | |
|  | **r *_s_*** | **p-value** | **r *_s_*** | **p-value** | **r *_s_*** | **p-value** | **r *_s_*** | **p-value** |
| **EQ** | 0.069 | 0.709 | -0.066 | 0.721 | -0.143 | 0.435 | -0.103 | 0.575 |
| cognitive empathy | 0.004 | 0.982 | -0.169 | 0.354 | -0.008 | 0.965 | -0.114 | 0.534 |
| emotional reactivity | 0.052 | 0.778 | -0.062 | 0.736 | -0.091 | 0.619 | -0.101 | 0.583 |
| social skills | -0.067 | 0.716 | 0.015 | 0.936 | -0.227 | 0.212 | -0.141 | 0.441 |
| empathic difficulties | 0.199 | 0.274 | -0.094 | 0.610 | 0.021 | 0.911 | 0.024 | 0.897 |
| **RMET** | 0.100 | 0.585 | 0.160 | 0.382 | 0.116 | 0.527 | 0.147 | 0.421 |
| **Laughter authenticity discrimination** | -0.105 | 0.568 | 0.002 | 0.991 | 0.257 | 0.156 | 0.061 | 0.739 |
|  | **P200** | | | | | | | |
|  | **Authentic laughter** | | **Acted laughter** | | **Authentic crying** | | **Acted crying** | |
|  | **r *_s_*** | **p-value** | **r *_s_*** | **p-value** | **r *_s_*** | **p-value** | **r *_s_*** | **p-value** |
| **EQ** | 0.033 | 0.856 | -0.305 | 0.090 | 0.017 | 0.925 | 0.092 | 0.617 |
| cognitive empathy | 0.073 | 0.692 | -0.120 | 0.514 | 0.059 | 0.749 | 0.129 | 0.483 |
| emotional reactivity | -0.107 | 0.561 | **-0.361** | **0.042*** | -0.147 | 0.424 | -0.110 | 0.548 |
| social skills | 0.210 | 0.249 | -0.043 | 0.813 | 0.240 | 0.185 | 0.323 | 0.071 |
| empathic difficulties | -0.006 | 0.976 | -0.316 | 0.078 | -0.022 | 0.906 | -0.040 | 0.826 |
| **RMET** | 0.172 | 0.346 | -0.084 | 0.648 | 0.134 | 0.466 | 0.137 | 0.455 |
| **Laughter authenticity discrimination** | -0.075 | 0.685 | 0.233 | 0.200 | -0.258 | 0.153 | -0.340 | 0.057 |
|  | **LPC** | | | | | | | |
|  | **Authentic laughter** | | **Acted laughter** | | **Authentic crying** | | **Acted crying** | |
|  | **r *_s_*** | **p-value** | **r *_s_*** | **p-value** | **r *_s_*** | **p-value** | **r *_s_*** | **p-value** |
| **EQ** | 0.243 | 0.180 | 0.172 | 0.346 | 0.008 | 0.967 | 0.160 | 0.383 |
| cognitive empathy | 0.042 | 0.819 | 0.062 | 0.735 | -0.051 | 0.781 | 0.024 | 0.896 |
| emotional reactivity | 0.199 | 0.275 | -0.026 | 0.890 | -0.116 | 0.527 | 0.113 | 0.538 |
| social skills | 0.018 | 0.923 | 0.120 | 0.512 | -0.057 | 0.758 | -0.188 | 0.302 |
| empathic difficulties | **0.368** | **0.038*** | 0.269 | 0.137 | 0.186 | 0.309 | 0.337 | 0.059 |
| **RMET** | 0.238 | 0.190 | 0.116 | 0.526 | 0.111 | 0.545 | **0.401** | **0.023*** |
| **Laughter authenticity discrimination** | -0.278 | 0.123 | -0.360 | 0.043 | 0.010 | 0.955 | -0.185 | 0.310 |

**Table S3.** Correlations between trait empathy scores and authenticity discrimination index.

|  | **Authenticity discrimination index** | | | |
| --- | --- | --- | --- | --- |
|  | **Laughter** | | **Crying** | |
|  | **r *_s_*** | **p-value** | **r *_s_*** | **p-value** |
| **EQ** | -0.111 | 0.546 | -0.158 | 0.388 |
| cognitive empathy | -0.190 | 0.297 | -0.176 | 0.336 |
| emotional reactivity | -0.056 | 0.761 | -0.020 | 0.914 |
| social skills | 0.078 | 0.672 | -0.228 | 0.209 |
| empathic difficulties | -0.109 | 0.552 | -0.065 | 0.723 |
| **RMET** | -0.228 | 0.210 | -0.034 | 0.854 |

Table S4. Summary of average acoustic properties of the stimuli

| **Stimulus Type** | **Duration (ms)** | **Mean F0/Pitch (Hz)** | **Mean intensity (dB)** |  |
| --- | --- | --- | --- | --- |
|  |  |  |  |  |
|  | *(Mean* ± *SD)* | *(Mean* ± *SD)* | *(Mean ± SD)* |  |
| Authentic Laughter | 2399.94 ± 460.73 | 397.13 ± 90.62 | 66.10 ± .10 |  |
| Acted Laughter | 2248.89 ± 400.15 | 257.84 ± 60.26 | 66.04 ± .11 |  |
| Authentic Crying | 2685.44 ± 289.36 | 421.38 ± 57.04 | 63.40 ± 3.10 |  |
| Acted Crying | 2182.61 ± 351.48 | 368.62 ± 87.75 | 64.64 ± 6.93 |  |
| Neutral (vowel ‘ah’) | 2498.73 ± 292.08 | 182.13 ± 54.01 | 64.81 ± 0.04 |  |

Table S5. Repeated measure correlation coefficients (Rrm) between single-trial ERP amplitudes/latencies and acoustic stimuli properties, with their respective p-values.

|  | **pitch** | | **intensity** | | **duration** | |
| --- | --- | --- | --- | --- | --- | --- |
|  | **R*_rm_*** | ***p*-value** | **R*_rm_*** | ***p*-value** | **R*_rm_*** | ***p*-value** |
|  |  |  |  |  |  |  |
| **N100** | 0.025 | 0.055 | 0.006 | 0.619 | 0.008 | 0.518 |
| **P200** | -0.008 | 0.516 | 0.01 | 0.444 | 0.015 | 0.25 |
| **LPC** | 0.012 | 0.345 | 0.007 | 0.611 | -0.004 | 0.76 |
| **N100 latency** | 0.0135 | 0.322 | 0.022 | 0.322 | 0.002 | 0.899 |
| **P200 latency** | 0.018 | 0.195 | 0.016 | 0.234 | -0.01 | 0.451 |

Table S6. Repeated measure correlation coefficients (Rrm) between single-trial ERP amplitudes and acoustic stimuli properties, and their respective p-values. Significant correlation is underlined and signaled with an asterisk.

|  |  | **vocalisation type** | | | | | | | |  |
| --- | --- | --- | --- | --- | --- | --- | --- | --- | --- | --- |
|  |  | **real crying** | | **acted crying** | | **real laughing** | | **acted laughing** | |  |
| **Acoustic property** | **ERP measure** | Rrm | *p-value* | Rrm | *p-value* | Rrm | *p-value* | Rrm | *p-value* |  |
|  |  |  |  |  |  |  |  |  |  |  |
| **intensity** | **N100 amplitude** | -0.029 | 0.344 | 0.035 | 0.251 | 0.008 | 0.795 | 0.044 | 0.157 |  |
|  | **P200 amplitude** | -0.016 | 0.605 | 0.047 | 0.128 | -0.048 | 0.127 | 0.020 | 0.529 |  |
|  | **LPC amplitude** | -0.055 | 0.079 | 0.000 | 0.999 | 0.033 | 0.296 | 0.007 | 0.820 |  |
|  | **N100 latency** | 0.064 | 0.055 | 0.005 | 0.869 | 0.041 | 0.217 | -0.009 | 0.787 |  |
|  | **P200 latency** | 0.046 | 0.166 | 0.023 | 0.477 | -0.001 | 0.974 | -0.013 | 0.696 |  |
| **pitch** | **N100 amplitude** | 0.008 | 0.788 | 0.016 | 0.605 | -0.001 | 0.981 | 0.007 | 0.812 |  |
|  | **P200 amplitude** | 0.037 | 0.239 | -0.057 | 0.065 | -0.028 | 0.377 | -0.007 | 0.819 |  |
|  | **LPC amplitude** | -0.045 | 0.146 | 0.005 | 0.873 | 0.047 | 0.129 | 0.036 | 0.245 |  |
|  | **N100 latency** | 0.013 | 0.698 | -0.011 | 0.736 | 0.000 | 0.989 | 0.062 | 0.057 |  |
|  | **P200 latency** | -0.028 | 0.387 | 0.012 | 0.705 | 0.003 | 0.926 | 0.009 | 0.794 |  |
| **duration** | **N100 amplitude** | **-0.065** | **0.037*** | 0.026 | 0.401 | -0.010 | 0.754 | -0.030 | 0.334 |  |
|  | **P200 amplitude** | 0.005 | 0.862 | 0.005 | 0.877 | -0.007 | 0.814 | 0.035 | 0.262 |  |
|  | **LPC amplitude** | 0.008 | 0.799 | 0.013 | 0.681 | 0.028 | 0.367 | 0.004 | 0.886 |  |
|  | **N100 latency** | -0.030 | 0.366 | -0.018 | 0.590 | -0.001 | 0.988 | 0.011 | 0.733 |  |
|  | **P200 latency** | 0.002 | 0.949 | 0.002 | 0.957 | -0.014 | 0.673 | 0.023 | 0.487 |  |

**Table S7.** Summary of the main effects and interactions of authenticity and emotion valence on ERP latencies. Statistically significant effects (p <.05) are underlined and signaled with an asterisk.

|  | **Main effect/interaction** | **F(df)** | **p-value** | **ηp2** | **Comparison** |
| --- | --- | --- | --- | --- | --- |
| **N100** | Authenticity | 1.25 (29) | 0.272 | 0.041 | ~ |
|  | Emotion | 3.18 (29) | 0.085 | 0.099 | ~ |
|  | Auth*Emo | 0.57 (29) | 0.455 | 0.019 | ~ |
| **P200** | Authenticity | 0.48 (29) | 0.494 | 0.016 | ~ |
|  | **Emotion** | **4.52 (29)** | **0.042*** | **0.135** | **Laugh < Cry** |
|  | Auth*Emo | 0.008 (29) | 0.929 | 0.0003 | ~ |

Supplementary Text A

**Behavioural results**

**Authenticity ratings**

There was a main effect emotion, with laughter (*M* = 4.55, *SD* = 0.70) rated as more authentic than crying (*M* = 3.64, *SD* = 0.64) irrespective of their true authenticity, on authenticity ratings (F(1, 31) = 30.84, p < .001, η² = .50). Expectedly, the main effect of authenticity on authenticity ratings was also significant, with acted vocalisations (*M* = 3.53, *SD* = 0.55) rated as less authentic than authentic vocalisations (*M* = 4.66, *SD* = 0.72), irrespective of the emotion produced (F(1, 31) = 60.22, p < .001, η² = .66). There was no significant interaction between emotion type and authenticity (F(1, 31) = 0.41, p = .525, η² = .013; see Fig. S1).

**Emotional contagion and arousal ratings**

In terms of emotional contagion, there was a main effect of emotion (F(1, 26) = 23.76, p < .001, η² = .48), with laughter (*M* = 4.26, *SD* = 0.80) rated as more contagious than crying (*M* = 3.46, *SD* = 0.83), and a main effect of authenticity (F(1, 26) = 76.05, p < .001, η² = .74), with authentic vocalisations (*M* = 4.37, *SD* =.80) rated as more contagious than acted ones (*M* = 3.34, *SD* = 0.72). The emotion by authenticity interaction was not significant (F(1, 26) = 3.75, p = .063, η² = 0.13; see Fig. S1).

In terms of arousal ratings, there was a main effect emotion (F(1, 24) = 47.58, p < .001, η² = .66), with laughter associated with higher ratings (*M* = 4.20, *SD* = .89) than crying (*M* = 2.97, *SD* = .91), and authenticity (F(1, 24) = 67.69, p < .001, η² = .74), with higher arousal ratings in response to authentic (*M* = 4.14, *SD* = 0.93) than acted vocalisations (*M* = 3.03, *SD* = 0.76). The interaction between emotion and authenticity was not significant (F(1, 24) = 3.49, p = .073, η² = 0.13; see Fig. S1).

Supplementary Text B

**Inter subject variability in mood, working memory, and psychopathology**

To characterize inter-subject variability in mood, working memory and psychopathology – which can affect task compliance and performance - subjects executed the Positive and Negative Affect Schedule (PANAS: Watson *et al.*,1988; Portuguese version: Galinha and Pais-Ribeiro, 2005) for mood (Positive Affect Score: mean M = 42.8, SD = 9.06; Negative Affect Score: M = 30.3, SD = 10.27), the Working Memory (WM) Index of the Weschler Adult Intelligence Scale – Third Edition (WAIS – III: Weschler, 2008) for WM (WM Index: M = 19.5, SD = 3.46), and the Brief Symptom Inventory (BSI: Derogatis, 1983; Portuguese version: Canavarro, 2007) (Global Severity Index GSI: M = 0.73, SD = 0.51) for psychopathology. The BSI is used to identify self-reported clinically relevant psychological symptoms in adolescents and adults. It consists of 53 items covering nine symptom dimensions: Somatization, Obsession-Compulsion, Interpersonal Sensitivity, Depression, Anxiety, Hostility, Phobic anxiety, Paranoid ideation and Psychoticism; and three global indices of distress: Global Severity Index, Positive Symptom Distress Index, and Positive Symptom Total. The global indices measure current or past level of symptomatology, intensity of symptoms, and number of reported symptoms, respectively.

We considered any participant with a mean questionnaire score and ERP peak amplitudes 1.5 times above or below interquartile range (calculated across participants) to be an outlier to be removed from the analysis. Three participants were outliers in terms of PANAS negative affect scores; but as none of the three were outliers in terms of ERP peaks, they were not excluded.

Supplementary Text C

**Potential mediating factors**

As outlined in the introduction, auditory ERP components, especially N100 and P200, are sensitive to changes in low-level acoustic properties of stimuli. At the same time, the acoustic properties of vocalisations mediate recognition of their authenticity, as we reported previously (Anikin and Lima, 2018). Here, we explore whether changes in ERPs are driven by acoustic properties, which mediate authenticity recognition.

Firstly, we checked whether acoustic properties are associated with our independent variables (emotion and authenticity). Mean intensity, pitch, and duration (See Table S4 and Fig. S2) did not meet the assumption of normality and were therefore analysed using a non-parametric Kruskal-Wallis H test. We found that there was a statistically significant difference in mean intensity (*χ2*(3) = 33.039, *p* < .001), pitch (*χ2*(3) = 27.668, *p* = .001) and duration (*χ2*(3) = 14.228, *p* = .003) between experimental conditions. We followed this up with pairwise, Bonferroni-corrected Mann-Whitney tests. In terms of intensity, authentic crying was associated with lower intensity than both acted (p = .002) and authentic laughter (p < .001), and acted crying had lower intensity than both acted (p = .003) and authentic laughter (p < .001). In terms of pitch, acted laughter stimuli had lower pitch than acted crying (p = .008) authentic laughter (p < .001) and acted crying (p < .001). In terms of duration, acted laughter stimuli were of shorter duration than authentic crying (p = .006) and acted crying stimuli were of shorter duration than authentic crying (p = .008).

Next, we correlated acoustic properties with ERP amplitudes/latencies using a repeated-measures correlation technique *(rmcorr* package; (10)). We did this in two ways. In the first approach, we have put all available trials, from all participants, and from all conditions, into one model (see Table S5). Second approach involved putting all available trials, from all participants, but we have run one model per experimental condition. The results of this approach are shown in Table S6. Only the second approach showed a significant correlation between overall stimulus duration and N100 amplitude; however, we do not consider this as a confound given the early latency of this ERP component. The correlations between ERP amplitudes and intensity/pitch for three representative participants are also plotted in Fig. S3.

Supplementary Text D

**The ERP electrode and time-window choice justification**

The time intervals and electrodes subjected to statistical analysis were selected on the basis of subject-averaged ERP waveforms and topographic maps, collapsed across all experimental conditions to avoid bias (Luck and Gaspelin, 2017). Firstly, electrodes of interest were selected based on the inspection condition-averaged topography. The electrodes were then collapsed and single-subject ERPs (along with a grand-averaged ERP) were plotted to aid selection of a time window that would capture all individual peaks. See Figure S4 for topography and ERP plots that were used to make these decisions. The following time windows and electrode clusters were used:

N100: 80-200ms, electrodes: C1, C2, C3, C4, Cz, CP1, CP2, CP3 and CPz

P200: 180-350ms, electrodes Cz and FCz.

LPC: 500 – 998 ms, electrodes: PO3, PO4, PO7, PO8, POz, O1, O2, and Oz.

To increase precision of the measurement, the mean N100 and P200 amplitudes were measured between the peaks’ onset and offset, defined (on a participant basis) as the time point at which the amplitude reached 50% of the local peak on its either side (fractional peak latency measure; 10, 11). Measuring voltage at a single point in time (peak) is prone to noise, and although measuring the mean amplitude in the time window of interest is a superior method, it can be biased by using an inappropriate measurement window (Luck, 2014). The approach above is a variation of a mean around the peak approach and overcomes these problems. If the peaks could not be found for a specific participant/condition, the mean voltage within the time window of interest was entered into the analysis to avoid missing data. The LPC was measured as an average activity within the time window of interest.
